# It Requires More Than Intelligence to Solve Consequential World Problems

**DOI:** 10.3390/jintelligence9030038

**Published:** 2021-07-19

**Authors:** Joachim Funke

**Affiliations:** Psychologisches Institut, Universität Heidelberg, Hauptstr. 47, D-69117 Heidelberg, Germany; joachim.funke@psychologie.uni-heidelberg.de

**Keywords:** grand societal challenges, Sustainable Development Goals, complex problems, consequential world problems

## Abstract

What are consequential world problems? As “grand societal challenges”, one might define them as problems that affect a large number of people, perhaps even the entire planet, including problems such as climate change, distributive justice, world peace, world nutrition, clean air and clean water, access to education, and many more. The “Sustainable Development Goals”, compiled by the United Nations, represent a collection of such global problems. From my point of view, these problems can be seen as complex. Such complex problems are characterized by the complexity, connectivity, dynamics, intransparency, and polytely of their underlying systems. These attributes require special competencies for dealing with the uncertainties of the given domains, e.g., critical thinking. My position is that it is not IQ, but complex problem-solving competencies for dealing with complex and dynamic situations, that is important for handling consequential global problems. These problems require system competencies, i.e., competencies that go beyond analytical intelligence, and comprise systems understanding as well as systems control. Complex problem solving is more than analytic intelligence.

## 1. Introduction

The editors of this Special Issue have asked me to respond to the statement: “How intelligence can be a solution to consequential world problems”. What exactly are consequential world problems? Why should intelligence (intelligent behavior) be relevant to their solutions? What other skills would help in solving global problems? The answer I give here to these questions is based on my personal opinion, rather than on an evidence-based review of the literature.

Who defines “consequential world problems”? It is not easy to scale and prioritize the challenges to mankind, because there are many different perspectives. Lomborg (2007) tried to define “the world’s biggest problems” in terms of costs and benefits. However, for me (as a German, remembering that the Nazis used the term “life unworthy of life” [in German: “lebensunwertes Leben”] to evaluate different qualities of life), this approach suffers from the issue of giving a monetary value to human life. I cannot follow the idea that you can compensate a human life for money, in line with the belief that “all lives are equal”.

How, then, to define global problems? As “grand societal challenges”, one might define them as problems that affect a large number of people, perhaps even the entire planet, including problems such as climate change, distributive justice, world peace, world nutrition, clean air and clean water, access to education, and many more.

## 2. The Challenges

The world around us presents many challenges to mankind—not only human-driven climate change but also the demand for food and water for a global population that may soon reach 8 billion people. Clean air and clean water are not widely available in large parts of the world, driving migration from failed states to more promising ones.

The United Nations have compiled 17 “Sustainable Development Goals” that represent a collection of such global problems. These goals are concerned with the survival of planet Earth and its inhabitants. They are the blueprint to achieve a better and more sustainable future for all. They address the global challenges we face, including poverty, inequality, climate change, environmental degradation, peace, and justice.

What is psychology’s answer to these grand societal challenges? The research on problem solving is my preferred perspective on these challenges. Problem solving is a processual view on intelligence (see, e.g., Sternberg 1982). This research arena has produced various different approaches for dealing with nonroutine situations.

## 3. Proposed Solution: The “General Problem Solver”

The “General Problem Solver” (Newell et al. 1959) was, at its time (in the 1950s and 1960s), a promise to solve all problems with an algorithm. However, that promise did not hold—as it turned out, it was more a kind of “special” problem solver that could deal with well-defined problems but not with ill-defined ones. Real-world (ill-defined, “wicked”) problems normally show a series of attributes that simple (well-defined) problems lack:

- There is no clearly defined goal;
- There is more than just one (“the one and only”) solution;
- Information needed for the solution has to be actively searched for;
- There are dynamics in the problem situation (one cannot wait forever for a solution).

Today’s version of this is called “artificial intelligence”. This again promises to solve problems of many different types, but again, there is some disillusion concerning AI’s abilities when considering “adversarial perturbations” (e.g., Metzen et al. 2017) and missing explanations from deep-learning “black-box” algorithms (e.g., Rudin 2019).

## 4. Dealing with Uncertainty: Solving Complex Problems

As a consequence of the limitations of problem-solving research focused on puzzles, towers, and other “toy problems”, the use of computer-simulated microworlds was proposed (e.g., Brehmer and Dörner 1993). The use of microworlds within a controlled laboratory environment allowed the introduction of attributes that characterize uncertain situations and complex problems (see Dörner and Funke 2017; Funke 2019). According to Funke (2019, p. 167), a complex problem shows the following features: (1) *complexity*, in the sense that many variables are involved; (2) *connectivity*, reflecting the fact that relationships exist between variables; (3) *intransparency*, referring to missing or inaccessible information important for the problem-solving process; (4) *dynamics*, in the sense of the possibility of changes of a given situation over time; and (5) *polytely* (from the Greek word ‘polytelos’, meaning many goals), referring to there being many goals and objectives that are possible and could be pursued.

How are complex problems different to complicated problems? For complicated problems, solutions exist, and experts are needed (and found); for complex problems, there are no experts available, because these problems are novel and have no known solution (see Kurtz and Snowden 2003).

Contrary to expectations, the successful handling of complex problems does not correlate with intelligence (as measured by classical tests of intelligence), according to early studies (for a review, see Kluwe et al. 1991; Wenke et al. 2005). However, Wittmann and Süß (1999) pointed out that these negative results might be due to a Brunswikian asymmetric relationship between the dependent variables (single-act criteria on the side of complex problems and multiple-act criteria on the side of intelligence).

Research with “minimally complex problems” (as they were developed within PISA 2012; see Csapó and Funke 2017; Funke and Greiff 2017) showed that scores from these tasks explained about 10% of the variance in grade point average beyond test intelligence (Wüstenberg et al. 2012). However, concerning the validity of the “minimally complex systems”, questions and doubts remain (see, e.g., Funke 2014; Funke et al. 2017). To explore and control minimally complex problems, strategies such as VOTAT (“vary one thing at a time”) may be helpful (Schoppek and Fischer 2017), but in real life, they cannot be recommended as a useful strategy. For example, you normally cannot vary the mood of your boss (or other potentially influential variables) to test the possible hypothesis that, while in a sad mood, she/he might give you more difficult tasks than when in a happy mood. In complex situations, simple variations of single factors are neither possible (because there are too many variables) nor conclusive (you cannot control the influence of other explanatory causes).

Systems competencies seem to be more important than intelligence (Funke et al. 2018). In the 21st century, skills such “critical thinking” (e.g., finding and evaluating relevant information; Halpern and Dunn 2021), understanding the complexities and dynamics of large systems, and dealing with uncertainty better represent the requirements of life than finding the correct answer to a number-series task (as tends to be requested in conventional tests of intelligence).

What are the cognitive proficiencies of systems thinking? Systems thinking involves a collection of cognitive and noncognitive features. It is a holistic approach, switching between a bird’s eye view and a detailed view, remaining calm and persistent in the pursuit of goals, and showing empathy for the acting persons, instead of focusing on simple cause–effect chains, with a preference for systemic thinking about loops with positive or negative feedbacks.

Based on the proposed model, I illustrate more in detail how system competencies could be applied in practice. For this purpose, I considered an important contemporary real-world problem, namely climate change. Analytical intelligence (based on rational choice assumptions) might argue that, with respect to the anticipated rising sea level and myself living far above sea level, there is no threat to me as a person. However, with empathy (as part of a broader understanding of intelligence), one could imagine the threat to people living on an island in the Pacific Ocean exposed to a rising sea level.

## 5. Ethical Values, Character Formation, and Wisdom

Our current concept of intelligence is missing an ethical dimension. During the education process of individuals, we do not solely teach facts and knowledge; we also teach values. The education process, in general, is a process of character formation. One of the long-term results of character formation can be seen in the development of wisdom. Tuchman (1984, p. 21) defined wisdom as: “the exercise of judgment acting on experience, common sense and available information.” Is wisdom the result of successful character formation?

In her recent review, Glück (2019, Table 16.1, p. 310) presented twelve definitions of wisdom. Only one of them mentions “values” explicitly, namely the “balance theory of wisdom” from Sternberg (1998). According to that theory, wise people know that different people can have different values. This idea of “value relativism” in wise people is also one of the five criteria for wisdom within the Berlin wisdom paradigm (see, e.g., Baltes and Staudinger 2000). However, to know that there are different perspectives on dilemmata does not imply that one has clear moral values—it is a kind of metaknowledge, free of any special content. Values allow for “sinn” (German, “meaning”) in life.

Similarly, Fischer (2015) argued for a context-free view of wisdom and saw it as “independent of one’s values and context.” On the other hand, Fischer collected 12 propositions that were commonly known to wise men from four different cultures (Socrates, Jesus, Confucius, and Buddha). These four wise individuals show parallels concerning certain wise content (e.g., Proposition 10: “Good people (and children) make good company”). Once again, there is no comment about the acquisition of these pieces of wisdom. Reading such “wise” propositions does not make us a wise person instantaneously. To become a wise person is a process that normally demands time and life experience. Wisdom should be one of the competencies of good leaders.

The University of Cambridge Institute for Sustainability Leadership (CISL), as reported by Visser et al. (2016), examined leadership theories and leadership development framed within the United Nations Sustainable Development Goals (SDGs), which were launched in September 2016. The CISL summarized the elements of a ‘good’ global leader into a model based on earlier research by Visser and Courtice (2011). This approach argued that a leader operating with a global perspective, and in a complex context, should have seven characteristics: capacity to be a systems thinker, proficiency in navigating complexity, open-minded, long-term thinker, interdisciplinary, inclusive, and globally conscious.

## 6. Conclusions

Complexity continues to increase, and creative solutions to complex problems are urgently needed (e.g., Mainzer 2009; Puccio 2017). To address societal needs, intelligence must be enriched by an ethical dimension. Sternberg (2019, 2021a) uses the term “adaptive” as a qualification for a new perspective of intelligent behavior. From my point of view, intelligence in all its forms is adaptive. The new perspective is on society and on the survival of mankind and therefore takes value into account. Here, “menschenbild” (our view on man) matters; the metaphors of man have shifted from “homo oeconomicus” (skeptically: Sen 1977) over “homo ignorans” (Hertwig and Engel 2016) to “homo curans” (man that cares; see, e.g., Tronto 2017). The deployment of “transformational intelligence” (Sternberg 2021b) also comes into play. To quote Fyodor Dostoyevsky (1821–1881): “It takes something more than intelligence to act intelligently” (from his book *Crime and Punishment*). This “something more” could be values and wisdom. “Values” adds to the noncognitive variables and “wisdom” to the cognitive variables. This could make an important difference.
